# Can intraurethral stimulation inhibit micturition reflex in normal female rats?

**DOI:** 10.1590/S1677-5538.IBJU.2015.0135

**Published:** 2016

**Authors:** Tian Yu, Limin Liao, Jean Jacques Wyndaele

**Affiliations:** 1 Department translational neurosciences, Laboratory Urology, University of Antwerp, Faculty GGW, Belgium;; 2 Department of Urology, University Hopsital Antwerp, Antwerp, Belgium;; 3 Department of Urology, China Rehabilitation Research Center, Capital Medical University, Beijing, China;; 4 Department of Urology, Capital Medical University, Beijing, China

**Keywords:** Urination, Urodynamics, Rats, Urinary Bladder

## Abstract

**Objective:**

The study was designed to determine the effect of low frequency (2.5Hz) intraurethral electrical stimulation on bladder capacity and maximum voiding pressures.

**Materials and Methods:**

The experiments were conducted in 15 virgin female Sprague-Dawley rats (220–250g). The animals were anesthetized by intraperitoneal injection of urethane (1.5g/kg). Animal care and experimental procedures were reviewed and approved by the Institutional Animal Care and Use Committee of Antwerp University (code: 2013-50). Unipolar square pulses of 0.06mA were used to stimulate urethra at frequency of 2.5Hz (0.2ms pulse width) in order to evaluate the ability of intraurethral stimulation to inhibit bladder contractions. Continuous stimulation and intermittent stimulation with 5sec ‘‘on’’ and 5sec ‘‘off’’ duty cycle were applied during repeated saline cystometrograms (CMGs). Maximum voiding pressures (MVP) and bladder capacity were investigated to determine the inhibitory effect on bladder contraction induced by intraurethral stimulation.

**Results:**

The continuous stimulation and intermittent stimulation significantly (p<0.05) decreased MVP and increased bladder capacity. There was no significant difference in MVP and bladder capacity between continuous and intermittent stimulation group.

**Conclusions:**

The present results suggest that 2.5Hz continuous and intermittent intraurethral stimulation can inhibit micturition reflex, decrease MVP and increase bladder capacity. There was no significant difference in MVP and bladder capacity between continuous and intermittent stimulation group.

## INTRODUCTION

Neurogenic bladder (NB) dysfunction results from spinal cord injury (SCI), and is associated with neurogenic detrusor overactivity and detrusor sphincter dyssynergia. Complications secondary to urinary dysfunction, e.g. frequent urinary tract infections, vesicoureteral reflux, can lead to upper urinary tract damage, and ultimately to renal failure (1). Currently, clean intermittent catheterization with concomitant anticholinergic medication is the most common therapy of bladder management for most individuals with SCI (2). However, anticholinergic side effects include dry mouth, constipation, blurred vision and drowsiness. Anticholinergic medication also has a possibility to cross the blood–brain barrier and impair cognitive function (3).

It is known that electrical stimulation of the pudendal nerve is an alternative approach to restore urinary function. Electrical stimulation of the pudendal nerve at 3-15Hz (4, 5) results in a robust inhibition of detrusor activity in persons with SCI. While high frequency stimulation (20-50Hz) (6) facilitates reflex bladder contractions. It is also known that activation of afferents in the sensory pudendal nerve can reflexively induce efferent firing in the pudendal nerve to elicit sphincter muscle contractions that in turn can induce pudendal afferent firing via a motor–sensory coupling (7).

Therefore, it seems logical to hypothesize that intraurethral stimulation, by electrical stimulation of the pudendal nerve, will inhibit the micturition reflex by activation of the pudendal afferent and efferent pathways. Therefore, two metal rings can be arranged on the catheter which is used thrice or more in one day to treat NB. However, to our knowledge this electrically evoked urethrovesical inhibitory reflex mechanism has never been examined in female rat. The experiments revealed an urethrovesical reflex that inhibits bladder contractions and increases bladder capacity. In the present study, we determined the effect of low frequency intraurethral electrical stimulation on bladder capacity and maximum voiding pressures. The outcome of intermittent and continuous stimulation was compared. This reflex could be evoked by devices used for neuromodulation to benefit a large population of patients suffering from NB or overactive bladder.

## MATERIALS AND METHODS

The experiments were conducted in 15 virgin female Sprague-Dawley rats (220-250g). The animals were anesthetized by intraperitoneal injection of urethane (1.5g/kg). Animal care and experimental procedures were reviewed and approved by the Institutional Animal Care and Use Committee of Antwerp University (code: 2013-50). A ventral midline abdominal incision was made, and the bladder was catheterized via bladder dome with PE-50 polyethylene catheter (Clay-Adams, Parsippany, New Jersey). The catheter was connected by a three-way valve to both a pressure transducer (Emka technologies) connected to a NE-1000 syringe pump (New Era Pump Systems, Farmingdale, New York), and an exit port to empty bladder. A BD Insyte-WTM intravenous catheter (20 gauges) was inserted transurethrally. Two coated platinum wires were fully inserted into the catheter. The wires were hold in place securely and the catheter was pulled away from the urethra. The anode and the cathode were longer than the catheter 10mm and 8mm, respectively. The urethral meatus was tied around the wires (silk 3/0) to prevent leakage and firm the electrodes.

Based on a previous study (8) and our preliminary test, unipolar square pulses of 0.06mA were used to stimulate urethra at frequency of 2.5Hz (0.2ms pulse width) to value the ability of intraurethral stimulation to inhibit bladder contractions. Three control cystometrograms (CMGs) were performed during saline infusion without stimulation to obtain the control/baseline bladder capacity and evaluate reproducibility. Then continuous stimulation and intermittent stimulation with 5sec ‘‘on’’ and 5sec ‘‘off’’ duty cycle were applied during repeated saline CMGs, generated by an ISO-STIM 01DPI stimulator (Tamm, Germany). The continuous stimulation and intermittent stimulation started from filling to voiding contraction (greater than 25cm H_2_O) finished.

Bladder activity was recorded by a pressure transducer-1290C, Hewlett-Packard GMBH, Boeblingen, Germany, an amplifier-monitor (Emka technologies) and WINDAQ® DI-710 data acquisition. The bladder was emptied after each CMG and a 5–10 min rest period was respected between CMGs to allow the bladder reflexes to recover. The rats were sacrificed by urethane overdose.

Statistical analysis was performed using SPSS software (version 16.0 for Windows, SPSS, Chicago, IL, USA). Maximum voiding pressures (MVP) and bladder capacity were investigated during CMGs with or without 2.5Hz stimulation to determine the inhibitory effect on bladder contraction induced by intraurethral stimulation. Repeated measurements in the same animal during the same experiment were averaged to avoid the large variation caused by individual animal differences. All reported values are means±SD. One-way Anova followed SNK-q was used to detect statistical significance. For all statistical tests, P<0.05 was considered significant.

## RESULTS

**Figure 1:**
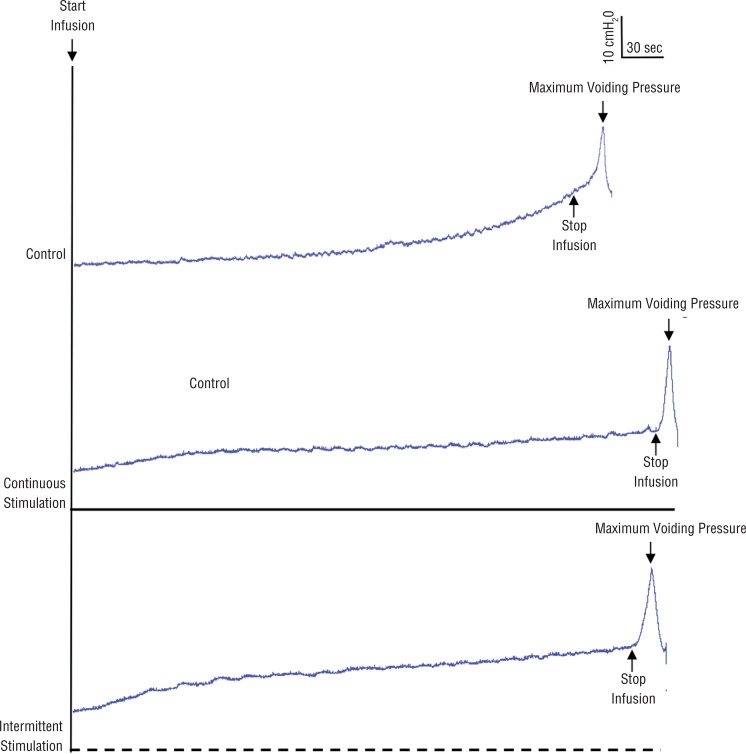
The continuous stimulation and intermittent stimulation (5sec on/5sec off) decreased maximum voiding pressure. Bladder capacity was increased by continuous stimulation and intermittent stimulation (5sec on/5sec off). The black bars under the traces indicate the stimulation duration. Infusion rate: 0.09mL/min.

**Figure 2:**
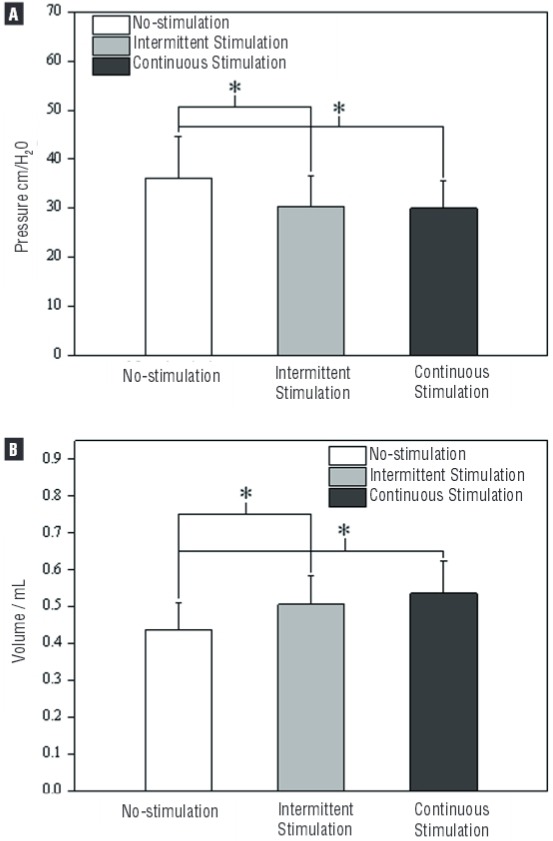
MVP (a) was significantly decreased (p<0.05) in continuous and intermittent stimulation (5sec on/5sec off) in 15 female rats when compared with no-stimulation group. Bladder capacity (b) was significantly increased by continuous stimulation and intermittent stimulation. There was no significant difference in MVP and bladder capacity between continuous stimulation group and intermittent stimulation group (a, b).

Continuous stimulation and intermittent stimulation significantly (p<0.05) decreased MVP from 36.2±8.5cmH_2_O to 30.0±5.6cmH_2_O and 30.4±6.2cmH_2_O, respectively (Figures 1 and 2a).

*Significantly (p<0.05) different from control.

Bladder capacity was maximally increased to 116±8.5% of the control/baseline capacity (0.438±0.074mL) during continuous stimulation. Intermittent stimulation with 5sec ‘‘on’’ and 5sec ‘‘off’’ duty cycle significantly increased bladder capacity to 123±12% of the control/baseline capacity (Figures 1 and 2b).

There was no significant difference in MVP and bladder capacity between continuous and intermittent stimulation groups (Figure-2).

## DISCUSSION

In the supra-SCI patients, control function in the cerebrum and the pons is blocked. Loss of supraspinal control leads to involuntary, reflexive bladder contractions and impaired coordination of the detrusor and sphincter system which can result in elevated bladder pressure during micturition and lead to structural damage of the bladder, vesicoureteral reflux, and renal insufficiency. Treatment for neurogenic bladder in SCI patients should fulfill three main objectives: low-pressure urine storage, low-pressure voiding, and adequate urine drainage. Clean intermittent catheterization with concomitant anticholinergic medication is currently the most common therapy of bladder management for most individuals with SCI (2). However, these drugs have dose-limiting side effects and may be insufficiently effective to restore continence in patients with severe hyperreflexia. When medical treatment is not satisfactory, surgical operations can be tried to increase the bladder capacity and compliance. The side-effects of medications and surgical complications have prompted us to seek new treatment modalities.

A relatively new alternative treatment is to activate the pudendal nerve after SCI, which can improve urine storage (9). Intraurethral stimulation not only activates the pudendal nerve, but also has advantages with its local effect and minimal invasion, which eliminates the systemic side effects caused by medication.

The afferent innervation of the lower urinary tract is carried in three sets of nerves: the pelvic and hypogastric nerves, which innervate the urinary bladder and proximal urethra, and the pudendal nerves, which innervate the mid-distal urethra and the external urethral sphincter (EUS) (10). The urethral length measured from the ureteral orifice to urethral meatus was 13.0 to 15.8mm in virgin female Sprague-Dawley rats (170-200g). The most proximal part of the striated muscle area was located 5.0 to 8.1mm from the ureteral orifice (11). The distance from the anode to the urethral meatus was 10mm in our experiments, meaning that the electrodes are positioned in the striated muscle area. The urethra was also dissected to confirm the position of electrodes in 3 rats in a preliminary test. So, the pudendal nerve could be directly modulated by intraurethral stimulation.

Previous studies proposed three possible mechanisms for the decrease of MVP and the increase of capacity evoked by intraurethral stimulation. The first proposal suggests that electrical stimulation of these somatic afferent fibers influence continence reflex pathways in the central nervous system (12). Secondly, low-frequency pudendal-afferent stimulation can evoke a robust reflex activation of hypogastric efferents (13). Therefore, low-frequency intraurethral stimulation suppresses bladder contractions that may arise from the activation of hypogastric efferent neurons, and subsequent synaptic and ganglionic inhibition of the parasympathetic-efferent neurons. Previous studies in the rat showed that pudendal sensory branch transection reduced bladder MVP (14), so the third possible mechanism can be the modulation of urethral afferent activity, which augments maximum bladder pressure and voiding efficiency.

This study provided pre-clinical evidence for designing a stimulator to stimulate the urethra intermittently instead of continuously while still achieving an inhibitory effect on bladder activity. Intermittent stimulation can reduce battery power consumption. In this study, at 5sec ‘‘on’’ and 5sec ‘‘off’’ ratio the inhibitory effect was also significantly (P<0.05) reduced when compared to the continuous stimulation. Thus, there is a trade-off between reducing the ‘‘on/off’’ ratio and maintaining the inhibitory effect.

The control experiments performed after short-time electrical stimulation reached baseline activity measured before electrical stimulation. This suggests the stimulation time can be extended to evaluate if it has a long-lasting post-stimulation inhibition function. In addition, the possible effects on delaying the progress of bladder fibrosis should be explored in SCI rat in our next study. Previous researches have demonstrated that somatic nerves stimulation increase bladder capacity in neurogenic bladder in animals and patients (14, 15), however, it was not clear if somatic nerves stimulation can decrease MVP.

Although clinical trials showed that the optimal stimulus parameters for neuromodulation therapy in human subjects are probably different from what was obtained in this animal study, the testing protocol and the results of this pre-clinical study will still provide very useful information for the design of a clinical trial that will use an intermittent stimulation strategy. Two Teflon wrapped metal rings can be arranged on the catheter which is used to drain urine from bladder every day in SCI patients to decrease bladder pressure or delay progress of bladder fibrosis (will be evaluated in next step). NB can be treated when intermittent catheterization is performed. Improving neuromodulation technology will benefit a large population of patients suffering from NB.

The present results suggest that 2.5Hz continues and intermittent intraurethral stimulation can inhibit micturition reflex, decrease MVP and increase bladder capacity. There was no significant difference in MVP and bladder capacity between continuous and intermittent stimulation group.
